# TaqMan Assays for Simultaneous Detection of *Bacillus anthracis* and *Bacillus cereus* biovar *anthracis*

**DOI:** 10.3390/pathogens9121074

**Published:** 2020-12-21

**Authors:** Diansy Zincke, Michael H. Norris, Odalis Cruz, Berzhan Kurmanov, W. Scott McGraw, David J. Daegling, John Krigbaum, Thi Thu Ha Hoang, Kamil Khanipov, Georgiy Golovko, Ted Hadfield, Jason K. Blackburn

**Affiliations:** 1 Spatial Epidemiology & Ecology Research Laboratory, Department of Geography, University of Florida, Gainesville, FL 32611, USA; dzincke@ufl.edu (D.Z.); mhnorris@ufl.edu (M.H.N.); odaliscc@ufl.edu (O.C.); berzhan.kurmanov@gmail.com (B.K.); thadfield@ufl.edu (T.H.); 2 Emerging Pathogens Institute, University of Florida, Gainesville, FL 32611, USA; 3 Department of Small Animal Clinical Sciences, College of Veterinary Medicine, University of Florida, Gainesville, FL 32611, USA; 4 Department of Anthropology, The Ohio State University, Columbus, OH 43210, USA; mcgraw.43@osu.edu; 5 Department of Anthropology, University of Florida, Gainesville, FL 32611, USA; daegling@ufl.edu (D.J.D.); krigbaum@ufl.edu (J.K.); 6 National Institute of Hygiene and Epidemiology, Hanoi 10000, Vietnam; htth@nihe.org.vn; 7 Department of Pharmacology & Toxicology, University of Texas Medical Branch, Galveston, TX 77555, USA; kakhanip@utmb.edu (K.K.); gegolovk@utmb.edu (G.G.)

**Keywords:** *Bacillus anthracis*, *Bacillus cereus* biovar *anthracis*, anthrax, multiplex, Ba-1, Africa

## Abstract

Anthrax is a worldwide zoonotic disease caused by the spore-forming bacterium *Bacillus anthracis*. Primarily a disease of herbivores, human infections often result from direct contact with contaminated animal products (cutaneous and inhalational anthrax) or through consumption of infected meat (gastrointestinal anthrax). The genetic near neighbor, *Bacillus cereus* biovar *anthracis* (Bcbva), causes an anthrax-like illness in the wildlife and livestock of west and central Africa due to the presence and expression of *B. anthracis*-specific virulence factors in this background. While Bcbva infections have not been reported in humans, a recent seroprevalence study detected Bcbva antibodies in the rural population around Taï National Park. This work describes the development of new TaqMan multiplex PCRs for the simultaneous detection of *B. anthracis* and Bcbva. The assays are designed to amplify Ba-1, *capB*, and *lef* markers in *B. anthracis* and genomic island IV (GI4), *capB*, and *lef* in Bcbva. Our assays allow for the rapid discrimination of *B. anthracis* and Bcbva and will provide insights into the molecular epidemiology of these two important pathogens that share an overlapping geographical range in west and central Africa.

## 1. Introduction

The Gram-positive spore producing *Bacillus anthracis* is the causative agent of anthrax, a worldwide zoonosis that primarily affects livestock and herbivores. Human infections are predominantly acquired through contact with or ingestion of infected animal products [1,2]. Cutaneous anthrax, the most common presentation in humans, is estimated to account for 20,000 cases annually and has a 20% mortality rate without treatment [3,4,5]. Another closely related member of the *B. cereus* group, *Bacillus cereus* biovar *anthracis* (Bcbva), has been reported to cause anthrax-like illness in wildlife and the livestock of west and central Africa [6,7,8,9,10,11]. Genotypically, Bcbva forms its own distinct clade and is more closely related to other members of the *B. cereus* group than to *B. anthracis* [12,13]. Phenotypically however, Bcbva shares some traits with *B. anthracis*, including the characteristic Medusa head appearance, the tenacious colony consistency, and the absence of beta-hemolysis [8,12]. Notably, Bcbva harbors the pXO1- and pXO2-like plasmids pBCXO1 and pBCXO2, which like their counterparts in *B. anthracis*, encode anthrax toxin (*pag*, protective antigen; *lef*, lethal factor; *cya*, edema factor), and the poly-γ-d-glutamate capsule, respectively [12,13]. In addition, the presence of chromosomal genomic islands, most of which are thus far unique to Bcbva, can be used as markers for specific detection of the pathogen [12,13]. While Bcbva anthrax-like infections have not been reported in humans, a recent seroprevalence study detected Bcbva-specific antibodies in the rural population around Taï National Park [14,15]. Improved diagnostics will be essential in uncovering and detecting cases in areas reporting Bcvba in bush meat or livestock.

Currently, no single assay exists for the rapid and simultaneous identification of these two pathogens that share an overlapping geographic distribution in western and central Africa [16,17,18,19,20]. Prompt and concurrent diagnosis is necessary in areas where both *B. anthracis* and Bcbva circulate and could further aid in monitoring the spread of Bcbva across its currently known geographical range. Our work here describes the development of two independent TaqMan multiplex PCR assays for the simultaneous identification of *B. anthracis* and Bcbva based on detection of unique chromosomal and plasmid markers. Specifically, the assay targeted the *lef* (pXO1) and *capB* (pXO2) virulence markers of *B. anthracis*, which are also present in the pXO1- and pXO2-like plasmids of Bcbva. The previously described chromosomal marker Ba-1 was first introduced for detection of *B. anthracis* and used for multiple outbreaks and for species confirmation ahead of genotyping [17,21]; the marker is routinely used to screen field and laboratory strains with high repeatability and is routinely confirmed with multi-locus variable tandem repeat analysis (MLVA) genotyping [17,21]. The Ba-1 marker appears to be unique to *B. anthracis* and encodes a hypothetical protein of unknown function in Ames (GenBank: AJH92142.1, Ames). For identification of Bcbva, a previously published assay for detection of genomic island IV (GI4) unique to this pathogen was adopted [12]. The assays were optimized across two different platforms, QuantStudio 7 and the LightCycler 2.0 (LC2), and a globally diverse panel of strains were tested for assay validation. These assays are easily adaptable across a range of qPCR platforms and may be useful for the diagnosis of *B. anthracis* and anthrax-toxin producing *B. cereus* such as Bcbva in areas with limited sequencing capabilities.

## 2. Materials and Methods

### 2.1. Bacterial Strains and Plasmids

For this study, a panel of 26 globally diverse *B. anthracis* strains that included representatives of each major lineage was selected from the Martin E. Hugh-Jones *Bacillus anthracis* collection curated at the University of Florida (Table 1). This collection well represents the global diversity and geography of *B. anthracis* [22], including several low passage wild type strains [23,24]. In addition, five Bcbva isolates from the bones and teeth of deceased monkeys collected from Taï National Park between 1994 and 2010 (unpublished data), were also tested along with various *Bacillus cereus* and *Bacillus thuringiensis* strains (both from BEI Resources Repository; www.beiresources.org). Globally, there are few Bcbva strains available in collections. Here, we tested a bone collection used for anthropological study. Briefly, to recover Bcbva, our lab tested bone fragments, teeth, or dried marrow from individual bones collected from Taï Forest, Côte d’Ivoire in West Africa representing *Cercocebus atys*, *Cercopithecus diana*, *Colobus polykomos*, *Piliocolobus badius* and *Procolobus verus* (Cercopithecoidea, Primates). Biological materials were bead beat using 0.1 mm beads for 5 min in 1X PBS 0.05% Tween-20 and centrifuged at 233× *g* for 2 min to remove large debris. The supernatant was centrifuged for 10 min at 3724× *g* to pellet bacterial spores. The pellet was cultured directly on sheep blood agar (SBA) or first incubated in 70% ethanol for 1 h then cultured on SBA. Non-hemolytic suspect colonies were sub-cultured for DNA extraction on SBA and confirmatory PCR (data not shown) was performed using singleplex qPCR assays targeting Ba-1, GI4, *lef* (lethal factor), and *capB* (capsule). For further confirmation, one of our isolates, Bc0001, was sequenced on the Illumina MiSeq platform and compared to the Bcbva type strain in GenBank (BioSample accession SAMN02603256). Additionally, a GI4 positive DNA control was constructed by cloning a 1297-bp region of Bcbva DNA encompassing GI4 sequences, into Blue Heron pUCminusMCS vector (pGI4) (Custom design, BlueHeron Biotech, Bothell, WA, USA).

### 2.2. Multiplex qPCR Assays

Previous TaqMan assays for the detection of *B. anthracis* [21] were adapted to the QuantStudio 7 Flex (Applied Biosystems, Foster City, CA, USA) and the LightCycler 2.0 (Roche, Indianapolis, IN, USA) instruments. For Bcbva, we adapted the GI4 marker and tested with plasmid targets (*capB* and *lef*) from Blackburn et al. [21]. The QuantStudio 7 assay consisted of a four-dye reaction for simultaneous detection of the plasmid markers *capB* and *lef*, as well as the chromosomal markers Ba-1 and GI4, unique to *B. anthracis* and Bcbva, respectively. PCR was carried out in a 20-µL reaction using 1 µL of template, 1 × PrimeTime Gene Expression Master Mix (1055772, IDT, Coralville, IA, USA), and primers and probes as described in Table 2. Cycling conditions were as follows: 95 °C for 3 min, and 45 cycles of 95 °C for 20 s and 60 °C for 30 s.

On the LC2 platform, two separate duplex assays were designed for simultaneous detection of FAM- and VIC-labeled probes (Table 3). Duplex one contained primers and probes for detection of Ba-1 and GI4 markers, species detection markers. Duplex two was used for detection of virulence markers *lef* and *capB* found in pXO1 and pXO2 plasmids (or pXO1- and pXO2-like), respectively. PCR was similarly carried out in a 20-µL reaction using 1 µL of template, 1x PrimeTime Gene Expression Master Mix (1055772, IDT, Coralville, IA, USA), and primers and probes as described in Table 3. Spectral overlap between fluorescent channels from the LC2 instrument were corrected by performing color compensation experiments using FAM and VIC probes according to the manufacturer’s instructions. The resulting color compensation file was applied to data obtained from the LC2 duplex assays. Cycling conditions were as follows: 95 °C for 3 min, and 45 cycles of 95 °C for 20 s and 60 °C for 30 s.

All assays were tested on all 26 *B. anthracis* strains using ~1.7 × 10^5^ genome equivalents (GE) of DNA (~1 ng). In addition, DNA from Bcbva, *Bacillus thuringiensis,* and *B. cereus* were included to test for specificity.

### 2.3. Limit of Detection of Multiplex Assays

To establish sensitivities, 10-fold serial dilutions of *B. anthracis* Ames (UF01106) DNA and a plasmid carrying GI4 sequences were tested in triplicate. Specifically, Ames DNA was used to establish limit of detection for the Ba-1, *lef*, and *capB* markers. pXO1 and pXO2 plasmid concentrations were estimated in Ames by the absolute quantification method using Ba-1 chromosomal marker as reference. To establish the sensitivity of the Island 4 marker, pGI4, a plasmid carrying GI4 sequences from Bcbva, was used as positive control for this marker. Sensitivities were established for each marker in the Quantstudio7 multiplex assay and both LC2 duplex assays.

### 2.4. Whole Genome Sequencing of Bcbva Isolate

Nextera XT DNA Library Preparation Kit was used to prepare a Whole Genome Sequencing library of Bc0001. The sample was barcoded using dual indexing with Nextera XT Index Kit v2. The library was quantified with a Qubit 3.0. Paired end sequencing was performed on an Illumina MiSeq. Quality filtration was performed on the samples to trim sequencing adapters, low quality and unknown nucleotides using CLC Genomics Workbench 12.0.2 “Trim Sequences” module. The files were then taxonomically classified using CLC Genomics Workbench 12.0.2 “Taxonomic Profiling” module against an optimized reference Microbial Reference Genome Database (June 2019) with all available *B. cereus* reference sequences additionally included. Additionally, the assembled genome and selected reference *Bacillus spp.* genomes underwent whole genome single nucleotide polymorphism (SNP) alignment with RAxML phylogenetic inference and bootstrapping using the PhAME software suite developed at Los Alamos National Labs [28]. The sequencing data are available in NCBI with BioSample accession SAMN15804163.

## 3. Results

### 3.1. Level of Detection of Duplex and Multiplex Assays

**Figure 1:**
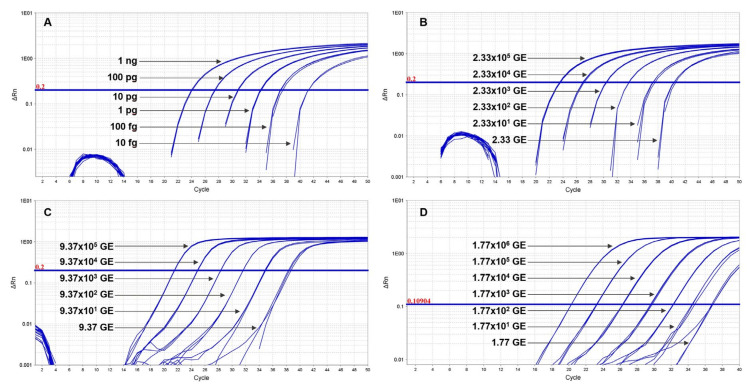
Sensitivity of TaqMan multiplex in QuantStudio. Ten-fold serial dilutions of Ames (Panels (**A**), (**B**) and (**C**)) or control plasmid carrying GI4 from Bcbva (Panel (**D**)) were tested in triplicate to establish the limit of detection of each primer pair and corresponding probe. The *B. anthracis* genome specific marker Ba-1 was consistently detected at 100 fg of genomic DNA, with sporadic amplification at the 10-fg level (Panel (**A**)). *capB* (Panels B) and *lef* (Panels (**C**)) targets were detected with as little as ~2 and 9 GE of pXO2 and pXO1, respectively, but could not be amplified at a lower 10-fold dilution (Panels (**B**) and (**C**)). GI4 was reliably detected with at least 17.7 GE of pGI4 (Panel (**D**)). The standard curve plots displayed *R*^2^ and slopes values as follows: Ba-1, *R*^2^ = 1, slope = −3.3793; *capB*, *R*^2^ = 0.9986, slope = −3.3337; *lef*, *R*^2^ = 1, slope = −3.3519; GI4, *R*^2^ = 0.9996, slope = −3.1776.

**Figure 2:**
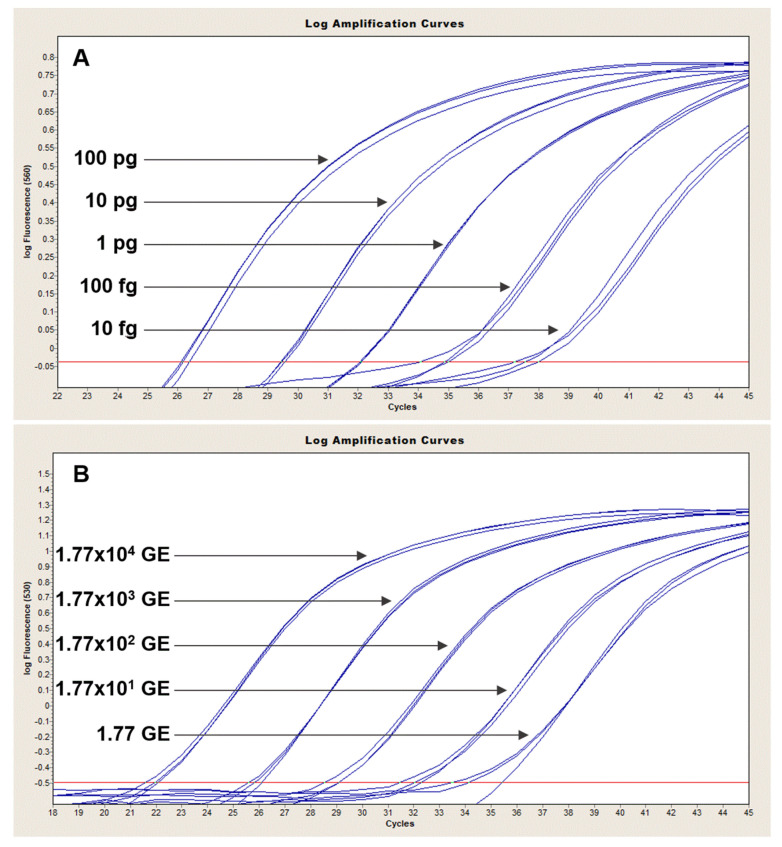
Sensitivity of TaqMan Ba-1-GI4 duplex in LightCycler 2.0. Ten-fold serial dilutions of Ames (Panel (**A**)) or control plasmid carrying GI4 from Bcbva (Panel (**B**)) were tested in triplicate to establish the limit of detection of *B. anthracis* Ba-1 and Bcbva GI4 chromosomal markers. Ba-1 displayed a sensitivity of 100 fg with inconsistent amplification at the 10-fg level (Panel (**A**)). About 17.7 copies of pGI4 were needed for reliable and reproducible amplification of GI4. The standard curve plots displayed *R*^2^ and slopes values as follows: Ba-1, *R*^2^ = 0.998, slope = −2.989; GI4, *R*^2^ = 0.9972, slope = −3.313.

**Figure 3:**
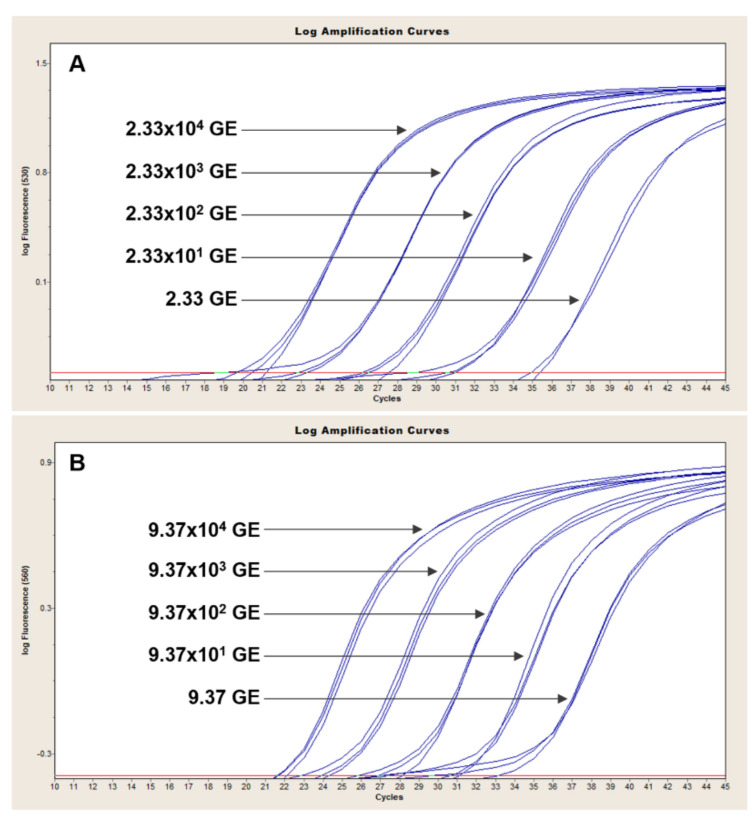
Sensitivity of TaqMan *capB* and *lef* duplex in LightCycler 2.0. Ten-fold serial dilutions of an Ames strain carrying pXO1 and pXO2 plasmids were tested in triplicate to establish the limit of detection of *capB* and *lef* markers. *capB* and *lef* were consistently detected with ~2 × 10^1^ and ~9 × 10^1^ GE of pXO2 (Panel (**A**)) and pXO1 (Panel (**B**)), respectively. The standard curve plots displayed *R*^2^ and slopes values as follows: *capB*, *R*^2^ = 0.976, slope = −3.4404; *lef*, *R*^2^ = 0.9594, slope = −3.486.

The Ba-1 marker displayed a limit of detection of 100 fg in both platforms with 10 fg-levels of DNA producing only sporadic amplification Figure 1A and Figure 2A). Similarly, the GI4 marker was reliably detected with 17.7 GE of pGI4 but not consistently at the 1.77 GE level (Figure 1D and Figure 2B). For the QuantStudio 7, *capB* and *lef* were consistently detected with ~2 and 9 GE of pXO2 and pXO1, respectively (Figure 1B,C), whereas reproducible detection on the LC2, necessitated at least ~2E1 and ~9E1 GE of pXO2 and pXO1, respectively (Figure 3A,B).

### 3.2. Four-Dye Multiplex in QuantStudio

**Figure 4:**
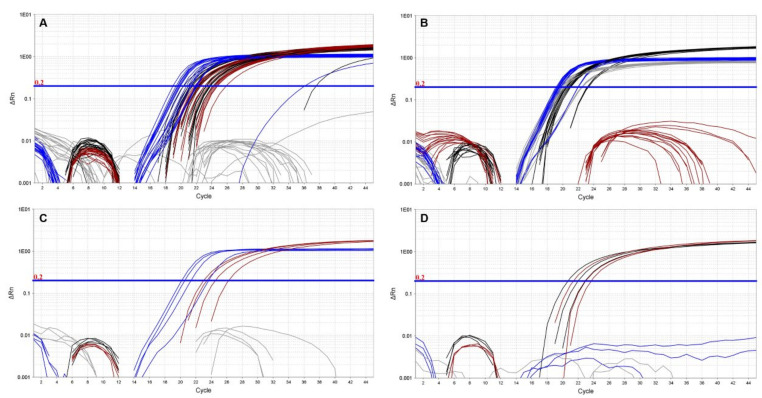
Evaluation of 4-color qPCR assay in QuantStudio. A collection of *B. anthracis* strains previously known to carry pXO1 and pXO2 produced consistent amplification of Ba-1 (red), *capB* (black) and *lef* (blue) markers (Panel (**A**)). The GI4 marker (grey) was specifically amplified in Bcbva backgrounds (Panel (**B**)) that were also strongly positive for *capB* (black) and *lef* (blue). Panel (**C**) illustrates amplification of Ba-1 (red) and *lef* (blue) markers in *capB*-negative *B. anthracis* isolates. Conversely, *lef*-negative *B. anthracis* only amplified Ba-1 (red) and *capB* (black) markers (Panel (**D**)).

The QuantStudio 7 multiplex assay is based on the simultaneous detection of *B. anthracis* and Bcbva-specific markers. We validated our assay with 26 *B. anthracis* strains of known marker status (any of the three markers; 19/26 positive for all three), as well as five Bcbva strains and closely related representatives of the *B. cereus* group. All 19 *B. anthracis* isolates known to be positive for *capB*, *lef* and Ba-1 markers produced clear and reproducible amplification curves of all three markers (Figure 4A and Table 4). The GI4 marker was specific to Bcbva (Figure 4B) and was not amplified in any other strains tested (Table 4). The five different Bcbva isolates tested showed strong amplification of *capB*, *lef* and GI4 while failing to amplify the *B. anthracis*-specific Ba-1 marker (Figure 4B). The specificity of *capB* and *lef* assay was illustrated with *capB*- and *lef*- negative strains. Specifically, the four different *B. anthracis capB* negative strains tested produced strong *lef* and Ba-1 signals (Figure 4C) whereas only Ba-1 and *capB* were amplified in *lef*-negative strains (Figure 4D).

### 3.3. Duplex Assays in LightCycler 2.0

**Figure 5:**
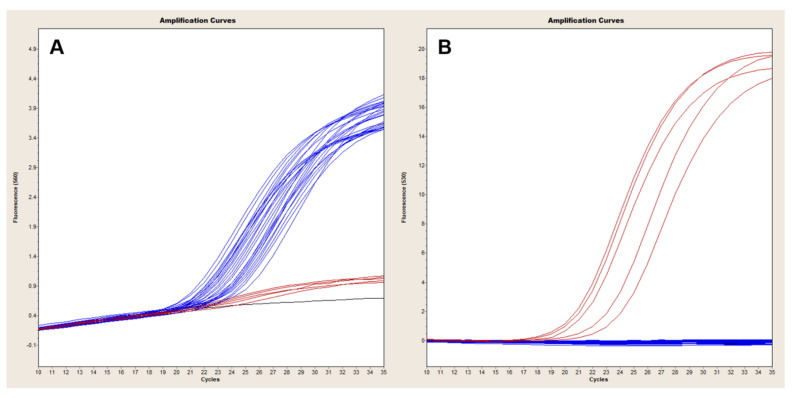
Evaluation of Ba-1-GI4 duplex PCR in LightCycler 2.0. A collection of *B. anthracis* and Bcbva strains were evaluated with the Ba-1 and GI4 TaqMan duplex PCR using 1 ng of genomic DNA. All *B. anthracis* strains tested (blue, panel (**A**)) showed clear amplification with the Ba-1 marker in the 560 channel, which detects signal from the VIC-labeled Ba-1 probe. Conversely, signal from GI4 was only observed for Bcbva (red, panel (**B**)) in the 530 channel, which detects signal from the FAM-labeled GI4 probe.

The LightCycler assays are based on the detection of chromosomal *B. anthracis* and Bcbva markers in one reaction and detection of plasmid markers in a second duplex reaction. Ba-1 signal was readily detected in all *B. anthracis* isolates tested (Figure 5A) and was specific up to 35 cycles of amplification with sporadic late cycle amplification appearing in non-Ba-1 backgrounds thereafter. Conversely, GI4 signal was only observed in Bcbva (Figure 5B) with low background signal for this marker observed in non-Bcbva isolates (Figure 5A).

**Figure 6:**
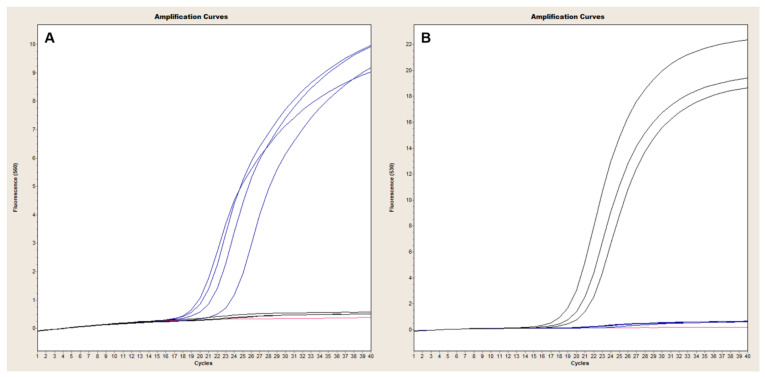
Evaluation of *capB-lef* duplex PCR in LightCycler 2.0. A collection of *B. anthracis* and Bcbva strains were evaluated with the *capB* and *lef* TaqMan duplex PCR using 1 ng of genomic DNA (Table 4). Amplification of *lef* in *capB*-negative *B. anthracis* strains is illustrated in Panel (**A**) (blue signal), with little to no *lef* background signal detected in isolates lacking pXO1 (Panel (**A**), black signal). Conversely, *capB* signal was readily detected in strains harboring pXO2 (Panel (**B**), black signal), but absent from *capB-negative* isolates (Panel (**B**), blue signal).

In the duplex assay for detection of *capB* (530 nm channel) and *lef* (560 nm channel), *capB*-negative strains exhibited strong signal in the 560 nm channel (Figure 6A, blue signal), corresponding to amplification of *lef*, that was absent from the 530 nm channel (Figure 6B). Similarly, *lef*-negative isolates produced signal only in the 530 nm channel corresponding to amplification of *capB* (Figure 6B, black signal). Both markers were readily detected in *B. anthracis* and Bcbva carrying pXO1, pXO2 and pXO1-like and pXO2-like plasmids (Table 4).

### 3.4. Specificity

To test specificity, various representatives of the *B. cereus* group were also tested in triplicate in both platforms. Ba-1, *capB* and GI4 markers, could not be detected in any these backgrounds (Table 4). The *lef* marker, however, produced reproducible late cycle amplification in *B. thuringiensis* serovar Kurstaki HD1. This strain carries a plasmid which displays 90% homology to pXO1 over an area that covers 16% of its sequence. *lef* was also strongly amplified in *B. cereus* G9241, which carries a pXO1-like plasmid harboring the genes for anthrax toxin subunits.

### 3.5. Whole Genome Sequencing of Bcbva Isolate Bc0001

**Figure 7:**
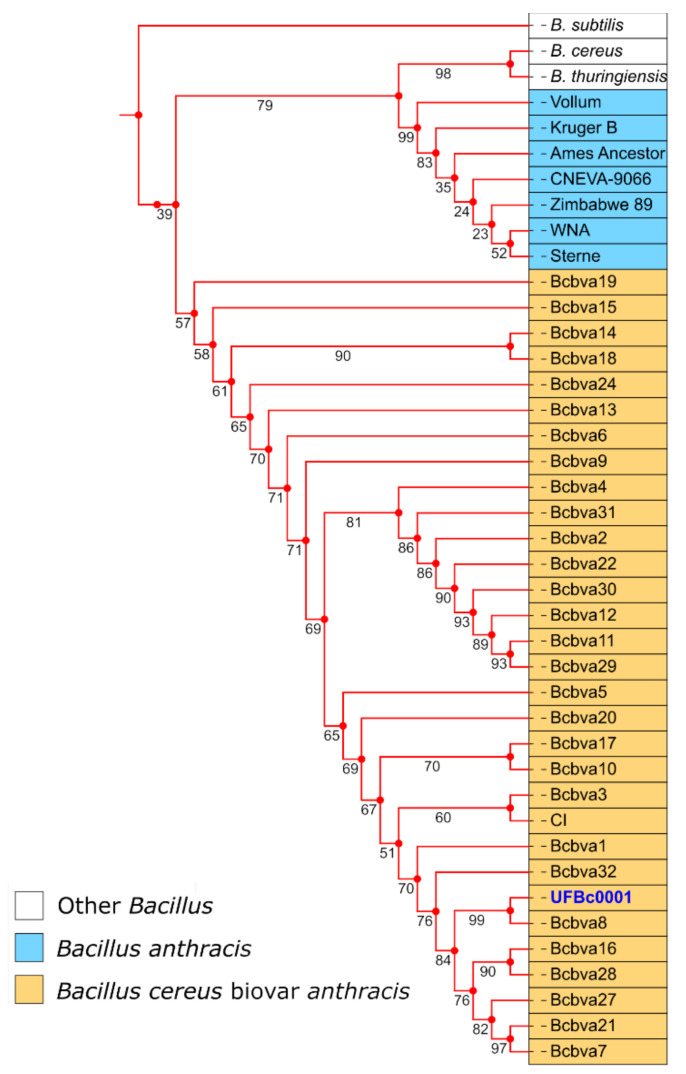
Phylogenetic tree illustrating relatedness of UFBc0001 (blue font) to Tai National Park isolates (orange boxes) and other members of the *Bacillus* genus (blue and white boxes) based on whole genome SNP alignments. Boot strapping values are shown on the tree branches.

Although the goal of this study was to develop qPCR assays for simultaneous detection of *B. anthracis* and Bcbva, we undertook whole genome sequencing of one of the Bcbva strains isolated in our lab to provide further confirmation of its identity. Isolate Bc0001 was sequenced on the Illumina MiSeq platform (BioSample accession SAMN15804163) and differed from the Bcbva type strain in GenBank (BioSample accession SAMN02603256) by 65 SNPs. Bc0001 contigs aligned with 99.58% identity to the Bcbva CI genome. Analysis by PATRIC (Pathosystems Resource Integration Center) estimated a genome length of 5,295,704 bp, an average G+C content of 35.21%, and a total of 5,640 protein coding sequences for Bc0001. A phylogenetic tree was constructed using various other Bcbva isolates from Taï National Park (Cote d’Ivoire) that were recently sequenced [10], as well as other members of the *Bacillus* genus. As expected, Bc0001 is closely related to other Bcbva isolates from Taï National Park, Cote d’Ivoire (Figure 7).

## 4. Discussion

The aim of this work was to develop and optimize a diagnostic assay for accurate, simultaneous, and differential detection of *B. anthracis,* the causative agent of anthrax, and Bcbva, which causes anthrax-like disease in non-human primates, wildlife, livestock, and possibly humans. Limited diagnostics have likely hindered the detection of human Bcbva infections. To that end, we have developed a four-color multiplex assay that can be readily applicable across 4-plex hydrolysis probes platforms (e.g., QuantStudio 3–12, StratageMx4000, BioRad iCycler IQ, ABI7900HT, etc.), and a two-reaction two-color assay for the LC2 capillary based format, which is an aging but commonly used platform worldwide (LightCycler). The LC2 assay is also adaptable to any 2-plex hydrolysis probes platforms. Our four-dye multiplex assay simultaneously detects *capB* and *lef* plasmid markers shared by both *B. anthracis* and Bcbva, as well as chromosomal markers that are unique to each pathogen. The capillary based assay requires two different 2-color reactions for detection of plasmid markers in one and chromosomal markers in the other. Although GI4 and Ba-1 markers serve as the primary difference between *B. anthracis* and Bcbva, it was important for us to have an assay that could ascertain the plasmid status of the strain and thus pathogenicity.

The GI4 marker was highly specific to Bcbva and could not be detected in any of the *B. cereus sensu lato* strains tested even at 50 cycles of amplification. Using the QuantStudio 7 multiplex assay, Ba-1 was specific to *B. anthracis* and absent from all Bcbva, *B. cereus,* and *B. thuringiensis* tested, with sporadic late cycle amplification of marker occurring in the LC2 duplex assays after 35 cycles of amplification. Although we tested specificity of the *capB* and *lef* markers in non-*B. anthracis B. cereus sensu lato*, the existence of *B. cereus* isolates with toxin and/or *cap* genes have been previously reported in humans [29,30,31,32,33,34]. Indeed, our assay was able to quickly detect the presence of *lef* in a *B. cereus* G9241 background known to carry pXO1 (Average C*_t_* = 16.22). Nonetheless, capsule and toxin markers were highly specific in *B. anthracis* yielding no amplification in strains lacking either *capB* or *lef*.

The TaqMan assays described in this work provide a rapid diagnostic tool for simultaneous detection of *B. anthracis* and Bcbva. Bcbva is an emerging pathogen that circulates in western and central Africa and a collective review of recent studies of both suggest overlap across much of the region [10,12,17,18,19,20,35]. Anthrax remains and important and underreported disease in this region and many of the classical microbiology techniques for differentiating *B. anthracis* from *B. cereus* do not work to differentiate Bcbva. Thus far reported in sylvatic animals, human impact is currently unknown. Bcbva is expected to be pathogenic in humans given that clinical manifestation in animals resemble classical anthrax. Recent work has identified serological tools that may further assist in human case detection [15]. Coupling those studies with these PCR assays in regional diagnostics labs could greatly improve our understanding of the epidemiology of anthrax (or anthrax like) disease caused by both pathogens. The Ba-1 and GI4 markers, thus far unique to *B. anthracis* and Bcbva respectively, are the basis for differentiation of these two pathogens in these qPCR assays. Aside from querying the organism specific genome markers, our assay can also establish the presence of *lef* and *capB* markers which are an integral part of the virulence machinery of *B. anthracis* and Bcbva.

## Figures and Tables

**Table 1:** Diversity panel of *Bacillus cereus* group used to validate multiplex PCR assays in this study.

A List/ Original ID	UF ID	Species	Strain	Lineage *	Strain Details
A0987	UF00175	*B. anthracis*		A.Br.005.006	Botswana
A0020	UF00552	*B. anthracis*	Ames	A3b	CAMR/Porton
A0537	UF00965	*B. anthracis*		A.Br.001/002	China
N/A	UF01137	*B. anthracis*	WNA	A1.a, A.Br.WNA	Colorado, domestic cow, 2012
A0897	UF00727	*B. anthracis*		A.Br.008/009	Ethiopia
A0389	UF00930	*B. anthracis*		A.Br.001/002	Indonesia
A0084	UF00980	*B. anthracis*	Vollum 1	A4, A.Br.Vollum	South Africa
A2075	UF01105	*B. anthracis*	Ames	A.Br.005.006	Tanzania
A0462	UF00738	*B. anthracis*	Ames	A3b	
A2017	UF01114	*B. anthracis*	Sterne	A.Br.001/002	Texas, white-tailed deer, 2009
A2076	UF01106	*B. anthracis*	Ames	A3b	Texas, white-tailed deer 2009
A2006	UF01096	*B. anthracis*	Vollum	A4, A.Br.Vollum	Texas, white-tailed deer 2009
A2064	UF01063	*B. anthracis*		WAG	Nigeria
A2067	UF01075	*B. anthracis*		WAG	Nigeria
	UF01052	*B. anthracis*		WAG	Nigeria
A0402	UF00926	*B. anthracis*		B.Br.CNEVA	France
A0333	UF00621	*B. anthracis*		B.Br.CNEVA	Germany
A0451	UF00438	*B. anthracis*		B.Br.001/002	Mozambique
A1088	UF00910	*B. anthracis*		B.Br.CNEVA	Poland
A0104	UF00839	*B. anthracis*		B.Br.001/002	South Africa
A1055	UF00603	*B. anthracis*		C.Br.A1055	USA
A0530	UF00878	*B. anthracis*		A.Br.005/006	Botswana
A1202	UF00049	*B. anthracis*			Argentina
HHG80	UF01135	*B. anthracis*			Etosha Natl Park (ENP), Namibia
A1073	UF00232	*B. anthracis*			Chile
A1075	UF00242	*B. anthracis*		A.Br.003/004	Chile
	Bc0001	Bcbva			Côte d’Ivoire (Taï Natl Park)
	Bc0002	Bcbva			Côte d’Ivoire (Taï)
	Bc0007	Bcbva			Côte d’Ivoire (Taï)
	Bc0009	Bcbva			Côte d’Ivoire (Taï)
	Bc0011	Bcbva			Côte d’Ivoire (Taï)
		*B. cereus*	FDA 5		
		*B. cereus*	Gibson 971		
		*B. cereus*	NRS 201		
		*B. cereus*	G9241		
		*B. thuringiensis*	Konkukian 97-27		
		*B. thuringiensis*	NRS 996		
		*B. thuringiensis*	AD-1		
		*B. thuringiensis*	HD-1		
		*B. thuringiensis*	Bt HD522		

* canSNP lineage/group as described in Sahl et al., Marston et al., Derzelle et al., Van Ert et al. [22,25,26,27].

**Table 2:** Gene targets, primers and probes used in multiplex on the QuantStudio 7 * platform to detect *B. anthracis* and *B. cereus biovar anthracis (*Bcbva) using real-time PCR.

Target	Oligo	Primer/Probe Sequence (5′–3′)	Final Concentration (nM)
Ba-1	Forward	GTACATCTTCTAGCTGTTGCAA	900
	Reverse	ACGTAGGAAGACCGTTGATTA	900
	Probe	VIC-CGTTGTTGTGTATTTG-MGB	250
*capB*	Forward	TAAGCCTGCGTTCTTCGTAAATG	600
	Reverse	GTTCCCAAATACGTAATGTTGATGAG	600
	Probe	NED-TTGCAGCGAATGAT-MGB	300
*lef*	Forward	CACTATCAACACTGGAGCGATTCT	600
	Reverse	AATTATGTCATCTTTCTTTGGCTCAA	600
	Probe	Cy5-AGCTGCAGATTCC-MGB	250
GI4	Forward	GGAGATATTAACAAGAGATGGATTGGA	700
	Reverse	CAGTAGGCTTGTCTGCTCTAATAAAATT	600
	Probe	FAM-ACATGCCAGCGTTTTTTGCCTCTACACA-BHQ1	150

* QuantStudio 7 excitation filter wavelengths in nm: 470 ± 15, 520 ± 10, 549.5 ± 10, 580 ± 10, 640 ± 10; emission filter wavelengths: 520 ± 15, 558 ± 12, 586.5 ± 10, 623 ± 10, 682 ± 10, 662 ± 10.

**Table 3:** Gene targets, primers and probes used in duplex PCR for the LightCycler 2.0 * platform to detect *B. anthracis* and Bcbva.

	Target	Oligo	Primer/Probe Sequence (5′–3′)	Final Concentration (nM)
Duplex 1	Ba-1	F-primer	GTACATCTTCTAGCTGTTGCAA	600
		R-primer	ACGTAGGAAGACCGTTGATTA	600
		Probe	VIC-CGTTGTTGTGTATTTG-MGB	250
	GI4	F-primer	GGAGATATTAACAAGAGATGGATTGGA	700
		R-primer	CAGTAGGCTTGTCTGCTCTAATAAAATT	600
		Probe	FAM-ACATGCCAGCGTTTTTTGCCTCTACACA-BHQ1	600
Duplex 2	*lef*	F-primer	CACTATCAACACTGGAGCGATTCT	400
		R-primer	AATTATGTCATCTTTCTTTGGCTCAA	400
		Probe	VIC-AGCTGCAGATTCC-MGB	250
	*capB*	F-primer	TAAGCCTGCGTTCTTCGTAAATG	600
		R-primer	GTTCCCAAATACGTAATGTTGATGAG	600
		Probe	FAM-TTGCAGCGAATGAT-MGB	250

* LightCycler 2.0 excitation by blue LED light source with maximum emission of 470 nm. Detection channels: 530, 560, 610, 640, 640, 705.

**Table 4:** Evaluation of *B. cereus* sensu lato panel with LC2 and QuantStudio 7 TaqMan assays. Average cycle thresholds are shown.

		LightCycler 2.0				QuantStudio 7			
Species	ID	Ba-1	*capB*	*lef*	GI4	Ba-1	*capB*	*lef*	GI4
Ba ^a^ Ames	UF00738	25.14	20.37	20.95	Und.	24.35	23.67	21.90	Und.
Ba Vollum 1	UF00980	24.69	32.12	32.45	Und.	25.85	37.52	36.08	Und.
Ba Ames	UF01105	25.49	20.06	20.49	Und.	23.09	22.25	20.60	Und.
Ba Ames	UF01106	26.17	20.46	20.89	Und.	24.69	23.73	21.92	Und.
Ba Vollum (A4)	UF01096	25.36	20.68	20.73	Und.	24.55	23.84	21.83	Und.
Ba WNA (A1.a)	UF01137	25.02	19.95	20.40	Und.	23.83	23.06	21.26	Und.
Ba	UF00175	25.31	20.84	20.77	Und.	24.74	24.13	21.91	Und.
Ba	UF01063	23.85	18.90	19.31	Und.	23.08	21.98	20.17	Und.
Ba	UF01075	24.36	20.06	20.33	Und.	24.73	23.73	21.89	Und.
Ba	UF01052	25.53	20.12	19.99	Und.	24.23	23.25	21.15	Und.
Ba	UF00049	25.93	20.11	19.80	Und.	24.40	23.31	21.02	Und.
Ba	UF01135	23.69	18.50	18.79	Und.	22.35	21.28	19.65	Und.
Ba	UF00438	23.82	17.15	17.84	Und.	21.75	20.31	18.89	Und.
Ba	UF00603	23.73	17.50	18.24	Und.	21.86	20.97	19.48	Und.
Ba	UF00727	23.83	18.26	17.94	Und.	22.40	22.17	19.86	Und.
Ba	UF00910	23.99	18.62	18.94	Und.	22.51	21.46	19.77	Und.
Ba	UF00926	23.39	18.01	18.34	Und.	21.98	21.25	19.42	Und.
Ba	UF00930	23.08	18.78	18.64	Und.	21.45	21.28	19.22	Und.
Ba	UF00965	23.69	17.97	18.48	Und.	22.09	20.99	19.49	Und.
Ba Sterne	UF01114	26.96	Und.	22.69	Und.	26.21	Und.	23.58	Und.
Ba	UF00839	23.79	Und.	18.93	Und.	23.39	Und.	20.47	Und.
Ba	UF00878	24.39	Und.	19.38	Und.	23.00	Und.	20.05	Und.
Ba	UF00232	25.35	Und.	20.35	Und.	24.59	Und.	21.26	Und.
Ba	UF00242	23.95	18.70	Und.	Und.	22.95	21.97	Und.	Und.
Ba	UF00502	24.59	19.71	Und.	Und.	23.65	22.89	Und.	Und.
Ba	UF00621	22.90	17.35	Und.	Und.	21.35	20.64	Und.	Und.
Bcbva	Bc0001	Und.	19.33	20.01	21.22	Und.	20.75	19.50	20.16
Bcbva	Bc0002	Und.	17.81	18.54	18.53	Und.	20.47	19.27	19.83
Bcbva	Bc0007	Und.	17.56	18.39	17.60	Und.	20.36	19.12	19.56
Bcbva	Bc0009	Und.	20.59	21.31	20.97	Und.	23.07	21.92	22.46
Bcbva	Bc0011	Und.	17.91	18.74	18.90	Und.	20.92	19.77	20.14
Bt ^b^ 97-27		Und.	Und.	Und.	Und.	Und.	Und.	Und.	Und.
Bt NRS 996		Und.	Und.	Und.	Und.	Und.	Und.	Und.	Und.
Bt AD-1		Und.	Und.	Und.	Und.	Und.	Und.	Und.	Und.
Bt HD-1		Und.	Und.	38.30	Und.	Und.	Und.	37.36	Und.
Bt HD522		Und.	Und.	Und.	Und.	Und.	Und.	Und.	Und.
Bc ^c^ FDA 5		Und.	Und.	Und.	Und.	Und.	Und.	Und.	Und.
Bc Gibson 971		Und.	Und.	Und.	Und.	Und.	Und.	Und.	Und.
Bc NRS 201		Und.	Und.	Und.	Und.	Und.	Und.	Und.	Und.
Bc G9241		Und.	Und.	20.12	Und.	Und.	Und.	16.22	Und.

^a^ Ba = *Bacillus anthracis*, ^b^ Bt = *Bacillus thuringiensis*, ^c^ Bc = *Bacillus cereus.* Und. = Undetermined, no amplification was detected.
